# Editorial: Cutaneous B-Cell Lymphomas

**DOI:** 10.3389/fonc.2020.619709

**Published:** 2020-12-09

**Authors:** Mauro Alaibac, Deniz Seckin, Pietro Quaglino

**Affiliations:** ^1^ Unit of Dermatology, University of Padua, Padua, Italy; ^2^ Unit of Dermatology, Baskent University, Ankara, Turkey; ^3^ Unit of Dermatology, University of Turin, Turin, Italy

**Keywords:** cutaneous B-cell lymphoma, skin cancer, B-cell, immunotherapy, immunosuppression

Primary cutaneous B-cell lymphomas (PCBCLs) are a relatively uncommon and heterogeneous group of lymphomas that present in the skin without evidence of systemic or extracutaneous disease at initial presentation (1). There have been significant changes in the diagnosis, staging, classification, and treatment of PCBCLs over the past ten years. These improvements are due in large part to advances in basic understanding of the disease and novel therapies, particularly those used to treat the aggressive subtype. Although the outcome is favorable for the majority of patients with PCBCLs, notably, patients with primary cutaneous marginal zone lymphoma (PCMZL) and primary cutaneous follicle center lymphoma (PCFCL), patients with primary cutaneous diffuse large B-cell lymphoma leg type (PCDLBCL-LT) have a worse prognosis and may exhibit treatment resistance (1). For these patients, an increasing number of compounds and therapeutic options have become available. However, at the same time, these options have increased complexity of management, as clinical trials to determine the best treatment regimen have been difficult to perform and no single standard of care exists. For PCDLBCL-LT chemotherapy, alone or combined with immunotherapy (e.g., rituximab), remains a gold standard for most patients and in most countries (Tadiotto Cicogna et al.). However, in recent years, a new wave of drugs have reached the market which may selectively target the critical pathways involved in the onset and progression of PCDLBCL-LT (e.g., inhibitors of the PD1/PDL1 pathway, inhibitors of the Bruton Tyrosine Kinase (BTK) pathway, mTOR inhibitors, BCL2 inhibitors) (Tadiotto Cicogna et al.).

It is now understood that the behavior of PCBCLs is influenced by an interplay between tumor cells and cells of the microenvironment and is mediated by host genetic factors. The articles in this Research Topic explore the biology and management of PCBCLs and evaluate the role of radiotherapy and emerging therapies, particularly in the more aggressive subtype (Tadiotto Cicogna et al.; Pileri et al.; Di Stefani et al.). Furthermore, the role of diagnostic methods and procedures, notably immunohistochemical and genetic investigations, bone marrow biopsy, computed tomography, and positron emission tomography scanning, are highlighted (Pileri et al.; Tadiotto Cicogna et al.). Finally, the relationship between immunosuppression and the development of certain subtypes of PCBCLs is discussed (Russo et al.).

In conclusion, this Research Topic collection exploring different aspects of PCBCLs, suggests novel points of discussion that will affect research in the near future. Moreover, it underlines our need to understand the biology of PCBCLs for better management and more appropriate therapeutic approaches. Overall, this research suggests optimistic expectations for the outcome of PCBCLs, particularly concerning PCDLBCL-LT, thanks to the ongoing development of novel, less toxic, and more efficacious treatment modalities.

## Author Contributions

MA wrote the manuscript. All authors contributed to the article and approved the submitted version.

## Conflict of Interest

The authors declare that the research was conducted in the absence of any commercial or financial relationships that could be construed as a potential conflict of interest.
